# Isolation and molecular characterization of *Cryptococcus* species isolated from pigeon nests and *Eucalyptus trees *

**DOI:** 10.29252/cmm.3.2.20

**Published:** 2017-06

**Authors:** A Kamari, A Sepahvand, R Mohammadi

**Affiliations:** 1 Department of Medical Parasitology and Mycology, School of Medicine, Isfahan University of Medical Sciences, Isfahan, Iran; 2 Razi Herbal Medicines Research Center, Lorestan University of Medical Sciences, Khorramabad, Iran; 3 Infectious Diseases and Tropical Medicine Research Center, Isfahan University of Medical Sciences, Isfahan, Iran

**Keywords:** *Cryptococcus gattii*, *Cryptococcus neoformans*, *Eucalyptus* trees, ITS sequencing, Pigeon nests

## Abstract

**Background and Purpose:**

*Cryptococcus* species are pathogenic and non-pathogenic basidiomycete yeasts that are found widely in the environment. Based on phenotypic methods, this genus has many species; however, its taxonomy is presently being re-evaluated by modern techniques. The *Cryptococcus* species complex includes two sibling taxa of *Cryptococcus neoformans* and *Cryptococcus gattii*. We aimed to investigate the possible distribution of *Cryptococcus *species in pigeon nests and *Eucalyptus* trees in Ilam, Iran, using molecular techniques.

**Materials and Methods:**

Two hundred and seventy-four specimens were collected from pigeon nests and *Eucalyptus* trees during 2016-2017. All the specimens were sub-cultured on Sabouraud Glucose Agar with chloramphenicol and bird seed agar. For molecular identification, the ITS15.8SITS2 rDNA region was amplified using the first and fourth internal transcribed spacer (ITS1 and ITS4, respectively) primers. The purified products were applied for cycle sequencing reactions in forward direction with ITS1 primer. The obtained results were analyzed with Chromas 2.3.

**Results:**

Thirty-three out of 186 cultures (17.7%) and 11 out of 88 cultures (12.5%) were positive among pigeon nest and *Eucalyptus* tree specimens, respectively. *Cryptococcus albidus* (17.2%), *C. albidus var. kuetzingii* (3.4%), *C. adeliensis* (3.4%), *C. uzbekistanensis* (3.4%), and *C. neoformans var. grubii* (3.4%) were isolated from pigeon nests, and *Cryptococcus* *adeliensis* (25%) was the only *Cryptococcus* species isolated from *Eucalyptus* trees.

**Conclusion:**

The presence of pigeons and *Eucalyptus* trees in the vicinity of some particular places such as rest homes and hospitals should be considered as a risk factor for the immunocompromised population.

## Introduction

*Cryptococcus *species are environmental basidiomycete yeasts that are found widely in the environment. The majority of the species live in the soil and are not pathogenic. According to the previous studies, the *Cryptococcus *genus includes more than 35 species [1, 2]; however, after more than 15 years of phenotypic and molecular studies, a proposal for a taxonomic revision was made. The genus *Cryptococcus *in its current concept encompasses the dimorphic yeasts *C. bacillisporus*, *C. decagattii*, *C. amylolentus*, *C. deneoformans*, *C. deuterogatttii*, *C. gattii*, *C. neoformans*, and *C. tetragattii*, as well as the filamentous species* C. luteus* and *C. depauperatus* [3]*.* Phylogenetic analysis revealed that *Cryptococcus* species complex includes two sibling taxa of *Cryptococcus gattii* and *Cryptococcus neoformans*. Both species are major of human and animal pathogens. *Cryptococcus neoformans *was first isolated by Sanfelice in 1894 in Italy from peach juice*. *The environmental origin of *C. neoformans* was obscure until Emmons reported pigeon nests and droppings as the main source of this fungal pathogen [4, 5]. Pigeons can even transmit* C. neoformans* on their feathers, beaks, and legs. The fungus is not the common flora of soil, and it is usually isolated from regions that are in contact with pigeons, turkeys, chickens, and other avian species [6, 7]. 

*Cryptococcus gattii*, formerly known as *Cryptococcus neoformans var gattii*, is another species of *Cryptococcus* found principally in the tropical and subtropical areas. It is a geographically limited fungus that has been found frequently in soil debris, especially regions with certain trees like oaks and *Eucalyptus* [8]. Northern Australia and Papua New Guinea have the highest incidence of *C. gattii* infection, but many cases of infection have also been reported from other areas including India, Vancouver Island in Canada, Brazil, and Washington State in the United States. 

Unlike *Cryptococcus neoformans*, that is remarkably associated with human immunodeficiency virus (HIV) infection or other immunodeficiency disorders, *C. gattii* can cause infection in healthy individuals, as well [9]. Soltani et al. [10] reported 3 out of 120 samples (2.5%) as the frequency of *C. neoformans *in towers of urban areas of Isfahan, Iran, in a period of nine months by using RapID Yeast Plus System and canavanine glycine bromothymol blue medium (CGB) test. Hedayati et al. [11] evaluated the isolation of *C. neoformans* from swallow (*Hirundo rustica*) excreta in two northern cities of Iran. They isolated *C. neoformans* from 5/97 (5.2%) of collected samples. Khosravi [12] sought for *C. neoformans* among 983 samples of pigeon droppings from various regions in northern Iran including Rasht, Ramsar, Babol, Sari, and Gorgan. They used phenotypic methods, namely culture on *Guizotia abyssinica *creatinine agar and CGB agar, and *C. neoformans* was isolated from 17.8% of the specimens. Badali et al. [13] isolated *C. neoformans* genotype AFLP1/VNI from a 49-year-old HIV-positive female in Sari, Iran, by sequencing the internal transcribed spacer (ITS) rDNA region. Salehei et al. [14] collected 156 samples of flowers, fruits, leaves, and barks of *Eucalyptus* trees and soil underneath *Eucalyptus *trees over a period of six months from various parts of Ahvaz, Iran. They used the traditional tests such as sub-culturing on Sabouraud Dextrose Agar, urease production, and growth at 37°C in the presence of capsule around yeasts using Indian ink preparation for the identification of *C. gattii*, but they could not isolate *C. gattii* from *Eucalyptus* trees and soil in Ahvaz. Due to the limited data on these potential pathogens in Ilam (a western province of Iran), the present study aimed to investigate the feasible distribution of *C. neoformans *and *C. gattii* in pigeon nests and *Eucalyptus* trees, respectively.

## Materials and Methods

**Sampling: **From November 2016 to March 2017, 274 specimens were obtained as follows:

**A)** **Pigeon nests:** One hundred and eighty-six samples were taken from pigeon nests in pet shops, houses of pigeon fanciers, and attics. Then, 15 g of pigeon droppings was collected by a sterile spade and put in ziplock bags. Laboratory processing of the samples was performed on the same day. The specimens were processed in accordance with Shields and Ajello method [7, 15]. Briefly, a suspension of each pigeon dropping was made in sterile saline solution 1:10 (w/v), and chloramphenicol (0.3 mg/ml) or streptomycin (2 mg/ml) (Sigma-Aldrich, Germany) was added to the suspension and mixed for 10 min and allowed to settle down for 40 min. Ten microliters of the supernatant of each suspension was added to Petri dishes containing *Guizotia abyssinica *agar (bird seed agar) (BIOMARK, India) and incubated at 30-32°C for three weeks. Coffee-colored colonies were considered *C. neoformans*. Each brown colony was examined microscopically using 10% KOH with methylene blue and Gram stain. Definitive identification of isolates was performed using DNA sequencing technique. 

**B)** ***Eucalyptus*** **trees: **Eighty-eight samples containing soil (30 g), leaves (20 g), woody debris (20 g), and tree bark (10 g) were put in sterile transport bags and stored for the following steps of the experiment. Four grams of each specimen was suspended in sterile water, vortexed, and allowed to settle down for about 10 min. Afterwards, 200 µl of each sample was transferred to Sabouraud Glucose Agar with chloramphenicol (Sigma-Aldrich, Germany), and all the yeast colonies were applied for sequencing [16, 17].

***Molecular identification***

**DNA extraction:** This process was conducted using boiling method [18, 19]. Briefly, 2-3 fresh colonies were suspended in 40 μl of double distilled water, boiled gently for 10-15 min, and then centrifuged for 7 min at 6000 rpm. The supernatant was used for polymerase chain reaction (PCR). 

**PCR:** The ITS15.8SITS2 rDNA region was amplified using ITS1 (5’TCC GTA GGT GAA CCT GCG G3’) and ITS4 (5’TCC TCC GCT TAT TGA TAT GC3’) primers [20]. The PCR conditions were as follows: denaturation of DNA at 95°C for 5 min, followed by 35 cycles of denaturation at 94°C for 30 s, annealing at 55°C for 45 s, and extension at 72°C for 1 min, with a final extension phase at 72°C for 7 min. The amplicons were purified by QIAquick PCR Purification Kit (Qiagen, USA). Seven microliters of each PCR product was run onto 1.5% agarose gel and electrophoresed in Tris–borate–EDTA buffer (90 mM boric acid, 2 mM 127 ethylenediaminetetraacetic acid, and 90 mM Tris) at 10 V/cm for 120 min, and 0.5 μg/ml of ethidium bromide (SigmaAldrich, Germany) was used for staining. 

**Sequencing:** The purified amplicons were applied for cycle sequencing reactions in forward direction (Bioneer, Korea) with ITS1 primer. The sequence results were analyzed with Chromas 2.3 (http://chromas.software.informer.com/2.3/). 

## Results

**Figure 1:**
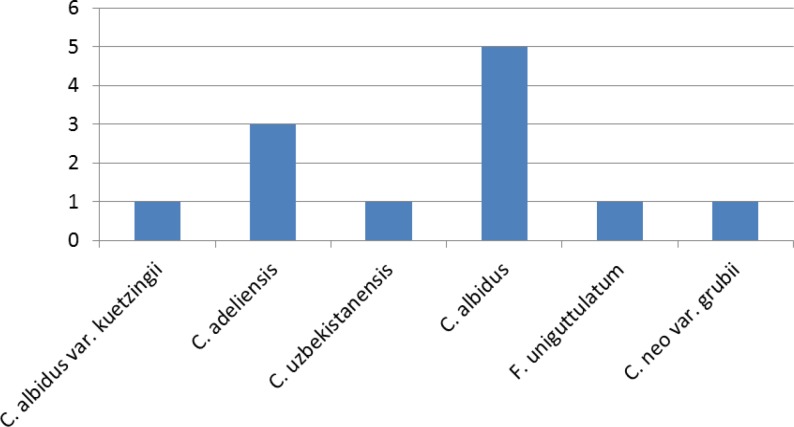
The frequencies of Cryptococcus species in the present study

Thirty-three out of 186 cultures (17.7%) and 11 out of 88 cultures (12.5%) were positive among pigeon nest and *Eucalyptus* tree specimens, respectively. Three positive samples of *Eucalyptus *trees and four positive cultures of pigeon nests had weak bands on agarose gel and were excluded from the investigation. Eighteen positive pigeon nest specimens (62.1%) were obtained from pet shops, 6 specimens (20.6%) from attics, and 5 specimens (17.2%) from houses of pigeon fanciers. Positive cultures of *Eucalyptus* trees belonged to leaves (no. 5; 62.5%), dust and debris around the trees (no. 2; 25%), and barks (no. 1; 12.5%). *Cryptococcus albidus* (*Naganishia* *albida*; 17.2%), *C. albidus var. kuetzingii* *(Naganishia uzbekistanensis; *3.4%), *C. adeliensis* *(N. adeliensis; *3.4%), *C. uzbekistanensis* (3.4%), and *C. neoformans var. grubii* (genotype AFLP1/VNI; 3.4%) were isolated from pigeon nests. *Cryptococcus* *adeliensis* (25%) was the only *Cryptococcus* species isolated from *Eucalyptus* trees. Figure 1 shows the frequency of *Cryptococcus* species in the present study. Table 1 presents all the yeasts isolated from both pigeon nests and *Eucalyptus *trees with their accession numbers.

**Table 1:** Yeast isolates obtained from pigeon nests and *Eucalyptus* trees and identified using polymerase chain reaction sequencing (rDNA ITS region)

**Pigeon Nests Isolates**			
**No.**	**Location**	**Isolate**	**Accession number**
1	Pet shops	*C. albidus var. kuetzingii (Naganishia uzbekistanensis)*	MG020688
2	Pet shops	*C. adeliensis (N. adeliensis)*	MG020689
3	Pet shops	*Hanseniaspora uvarum*	MG020690
4	Pet shops	*Cryptococcus uzbekistanensis*	MG020691
5	Pet shops	*C. albidus (N. albida)*	MG020692
6	Pet shops	*C. albidus (N. albida)*	MG020693
7	Pet shops	*Filobasidium uniguttulatum*	MG020694
8	Pet shops	*Debaryomyce shansenii*	MG020695
9	Pet shops	*Debaryomyces hansenii*	MG020696
10	Pet shops	*C. albidus (N. albida)*	MG020697
11	Pet shops	*C. albidus (N. albida)*	MG020710
12	Pet shops	*C. albidus (N. albida)*	MG020711
13	Pet shops	*Debaryomyces hansenii*	MG020712
14	Pet shops	*Rhodotorula mucilaginosa*	MG020720
15	Pet shops	*Candida albicans*	MG020713
16	Pet shops	*Meyerozyma guilliermondii*	MG020719
17	Pet shops	*Rhodotorula mucilaginosa*	MG020717
18	Pet shops	*Candida albicans*	MG020714
19	Attic	*C. neoformans var. grubii (genotype AFLP1/VNI)*	MG020698
20	Attic	*Debaryomyces hansenii*	MG020699
21	Attic	*Debaryomyces hansenii*	MG020700
22	Attic	*Debaryomyces hansenii*	MG020701
23	Attic	*Debaryomyces hansenii*	MG020702
24	Attic	*Candida albicans*	MG020703
25	Houses of pigeon fanciers	*Debaryomyces hansenii*	MG020704
26	Houses of pigeon fanciers	*Debaryomyces hansenii*	MG020705
27	Houses of pigeon fanciers	*Candida albicans*	MG020706
28	Houses of pigeon fanciers	*Meyerozyma guilliermondii*	MG020707
29	Houses of pigeon fanciers	*Candida albicans*	MG020715
	***Eucalyptus*** ** Tree Isolates**		
**No.**	**Location**	**Isolate**	**Accession number**
1	Leaves	*Trichosporon asahii*	MG020686
2	Leaves	*Rhodotorula mucilaginosa*	MG020687
3	Leaves	*C. adeliensis (N. adeliensis)*	MG020708
4	Leaves	*C. adeliensis (N. adeliensis)*	MG020709
5	Leaves	*Candida albicans*	MG020718
6	Bark	*Candida albicans*	MG020721
7	Dust and debris around the trees	*Candida albicans*	MG020722
8	Dust and debris around the trees	*Meyerozyma guilliermondii*	MG020716

## Discussion

*Cryptococcus neoformans* is a common yeast-like fungus in bird droppings, including pigeon, and soil contaminated with bird excrements. Although pigeon nests are considered as the natural habitat of *C. neoformans*, this species has been isolated from other sources like tree trunk hollows, bark, and decaying materials, as well [21]. In the present study, we isolated *Cryptococcus adeliensis* from *Eucalyptus* trees, but *C. neoformans* was not obtained from various parts of *Eucalyptus* trees. *C. adeliensis* was formerly described as a novel *Cryptococcus* species obtained from algae in Antarctica; this species is incapable of fermentation as is representative of the *Cryptococcus *species [22]. In the present investigation, we obtained three *C. adeliensis* isolates from pigeon droppings in pet shops (1 isolate) and the leaves of *Eucalyptus* trees (2 isolates). 

Rimek et al. [23] reported the first case of meningitis caused by *Cryptococcus adeliensis* in a German patient with acute myeloid leukemia. They completed phenotypic tests and identified isolates by sequencing the D1/D2, ITS 1, and ITS 2 regions of the 26S rDNA. In 2005, Tintelnot and Losert [24] isolated *C. adeliensis* from both clinical and environmental specimens. They isolated *C. adeliensis* from pigeon droppings collected from an urban recreation park (Tegel Lake in Berlin) and from a pigeon breeding facility near Hanover, Germany. They also isolated *C. adeliensis* from a lung biopsy of an adult male and from the oral cavity of an 8-year-old girl with HIV infection. *Cryptococcus adeliensis* grows in smooth and cream-colored colonies and is misidentified as *C. albidus* due to the high variability of its phenotypic characteristics. Therefore, molecular techniques such as sequence analysis for *C. albidus* clade are essential to identify *C. adeliensis*, as was performed in the present study. 

In 2016, Borhani and Rahimian [25] isolated *C. adeliensis* from cankers on stone fruits in Khorasan provinces, Iran. They showed stem canker caused by *C. adeliensis* is a newly emerged disease of fruit trees characteristically comparable to the bacterial canker disease [26]. *Cryptococcus* *albidus* was another species in the *Cryptococcus* genus isolated in this investigation. It is an uncommon cause of infection in humans and should be considered as a potential pathogenic agent of corneal ulcer. In 2015, Huang et al. [27] reported successful treatment of fungal keratitis due to *C . albidus* with amphotericin B. In 2014, Liu et al. [28] presented the first case of encephalitis due to *C. albidus* in an HIV patient. Intravenous fluconazole was applied for him but he died on day three. 

In 2015, Ragupathi et al. [29] reported the first case of *C. albidus* peritonitis in a patient infected with hepatitis C who was undergoing peritoneal dialysis due to renal failure. The patient was treated with amphotericin B; however, infections of non-*neoformans* cryptococcal species presented a clinical challenge because they are complicated to diagnose and treat. *Cryptococcus albidus* has five varieties, namely *C. albidus* var. *aerius*, *C. albidus* var. *albidus*, *C. albidus* var. *diffluens*, *C. albidus* var. *kuetzingii*, and *C. albidus* var. *ovalis*. One isolate of *C. albidus* var. *kuetzingii* was obtained from pet shops in the present study. 

*Cryptococcus uzbekistanensis* is a non-capsulated yeast that was first isolated from a desert soil sample from near Bukhara, Uzbekistan, in 1999 by Chernov et al. Afterwards, Fonseca et al. identified that this species causes glossy, smooth, cream to pinkish-cream colonies on yeast mold agar, with soft and butyrous texture. Review of veterinary and medical papers reveals that *C. uzbekistanensis* has never been isolated from an infection in humans or animals [30] until Powel et al. [31] reported the first case of cryptococcosis due to*C. uzbekistanensis* from the bone marrow of an immunocompromised patient with pancytopenia. Further, isolating *C. uzbekistanensis* from dust in US military samples has been reported in the Middle East [32]. We also isolated one case of *C. uzbekistanensis* from pigeon droppings in pet shops. We also reported an isolate of *Filobasidium uniguttulatum* from pet shops. *Filobasidium uniguttulatum*, is a teleomorphic fungus, which was first isolated in 1934 from an infected human nail, and then it was identified as *Eutorulopsis uniguttulata* [33]. On the basis of physiological and morphological similarities with *C. neoformans*, *Eutorulopsis uniguttulata* was renamed to *Cryptococcus neoformans* var*. uniguttulatus*, but with less capsule formation [34]. 

Phylogenetically, *F. uniguttulatum* is closer to* C. albidus* than to *C. neoformans* [35]. Among environmental strains, one case of *Cryptococcus neoformans var. grubii* was isolated from pigeon droppings in an attic. *Cryptococcus neoformans* is subdivided into two variants, that is, *C*. *neoformans* var. *neoformans *(var. D) and *C.* *neoformans* var. *grubii* (var. A)*.* The name “*grubii*” was chosen in honor of David Gruby, physician and scientist who lived in the 19th-century and first recognized and proved dermatophytosis.* Cryptococcus* *neoformans *var. *grubii* has a cosmopolitan distribution and is a fatal fungal pathogen among immunocompromised population [36].

In 2011, Hedayati et al. [11] isolated five *Cryptococcus* *neoformans *isolates from 97 enviro-nmental specimens from swallow excreta in Sari, Iran. All the yeasts were identified by colony characteristics on Niger seed agar (*Guizotia abyssinica*) and CGB medium. Khosravi et al. [12] used the same protocol for the isolation and identification of *Cryptococcus* species from pigeon (*Columba livia*) droppings in northern cities of Iran (Rasht, Ramsar, Babol, Sari, and Gorgan) and showed that all the isolates were *C. neoformans *var *neoformans*. Using CGB test and RapID Yeast Plus System, Soltani et al. [10] identified 3 out of 120 specimens as *Cryptococcus neoformans*. Out of 400 pigeon dropping specimens, Agha Kuchak Afshari et al. [37] found 20 (5%) samples positive for *C. neoformans* by sequence analysis of PCR products of the D1/D2 regions. In 2017, Dou et al. [38] characterized *C. neoformans* complex from environment in Beijing, China, using matrix-assisted laser desorption/ionization time-of-flight mass spectrometry (MALDI-TOF MS). They determined serotypes and mating types of isolates by specific primers, and restriction fragment length polymorphisms of URA5 (URA5-RFLP) were used for genotyping. In addition, multi-locus sequence typing (MLST) was performed for further identification. They identified 81 (18.6%) isolates of *C. neoformans* AFLP1/VNI; all the strains were serotype A. However, they did not isolate any other molecular types of *C. gattii* and *C. neoformans* strains. 

## Conclusion

We isolated and presented six species and variants of *Cryptococcus* and many other yeasts in pigeon dropping and *Eucalyptus* tree specimens. These potentially pathogenic yeasts can cause fatal fungal infections in immunocompromised individuals. The presence of pigeons and *Eucalyptus* trees close to some places such as rest homes and hospitals should be considered as a risk factor for this vulnerable population.
